# *Fusarium graminearum pyruvate dehydrogenase kinase 1* (*FgPDK1*) Is Critical for Conidiation, Mycelium Growth, and Pathogenicity

**DOI:** 10.1371/journal.pone.0158077

**Published:** 2016-06-24

**Authors:** Tao Gao, Jian Chen, Zhiqi Shi

**Affiliations:** 1 Institute of Food Quality and Safety, Jiangsu Academy of Agricultural Sciences, Nanjing, China; 2 Key Lab of Food Quality and Safety of Jiangsu Province-State Key Laboratory Breeding Base, Nanjing, China; 3 Key Laboratory of Control Technology and Standard for Agro-product Safety and Quality, Ministry of Agriculture, Nanjing, China; Seoul National University, REPUBLIC OF KOREA

## Abstract

Pyruvate dehydrogenase kinase (PDK) is an important mitochondrial enzyme that blocks the production of acetyl-CoA by selectively inhibiting the activity of pyruvate dehydrogenase (PDH) through phosphorylation. PDK is an effectively therapeutic target in cancer cells, but the physiological roles of PDK in phytopathogens are largely unknown. To address these gaps, a PDK gene (*FgPDK1*) was isolated from *Fusarium graminearum* that is an economically important pathogen infecting cereals. The deletion of *FgPDK1* in *F*. *graminearum* resulted in the increase in PDH activity, coinciding with several phenotypic defects, such as growth retardation, failure in perithecia and conidia production, and increase in pigment formation. The ΔFgPDK1 mutants showed enhanced sensitivity to osmotic stress and cell membrane-damaging agent. Physiological detection indicated that reactive oxygen species (ROS) accumulation and plasma membrane damage (indicated by PI staining, lipid peroxidation, and electrolyte leakage) occurred in ΔFgPDK1 mutants. The deletion of *FgPDK1* also prohibited the production of deoxynivalenol (DON) and pathogenicity of *F*. *graminearum*, which may resulted from the decrease in the expression of *Tri6*. Taken together, this study firstly identified the vital roles of *FgPDK1* in the development of phytopathogen *F*. *graminearum*, which may provide a potentially novel clue for target-directed development of agricultural fungicides.

## Introduction

Pyruvate, the end-product of glycolysis, is an important branch point in the sugar metabolism of most organisms [1]. Pyruvate can be metabolized by pytruvate dehydrogenase complex (PDC) that is composed of pyruvate dehydrogenase (PDH, E1), dihydrolipoamide acetyltransferase (E2), and dihydrolipoamide dehydrogenase (E3). PDC converts pyruvate into acetyl-CoA by a process called pyruvate decarboxylation. This multi-enzyme complex is related structurally and functionally to the oxoglutarate dehydrogenase and branched-chain oxoacid dehydrogenase multi-enzyme complexes. PDC activity is primarily regulated through phosphorylation (inhibition) and dephosphorylation (activation) of PDH on serine residues 293, 300, and 232 of the E1*α* subunit [2]. In mammals, the entrance of pyruvate into the tricarboxylic acid cycle is strictly regulated by the activity of the PDC. Pyruvate dehydrogenase kinase (PDK) is a mitochondrial enzyme that can result in the selective inhibition of PDH activity through phosphorylation and decrease the transition from glucose oxidation to acetyl-CoA production by the tricarboxylic acid (TCA) cycle, which further causes a bioenergetically disadvantageous increase in glycolysis, relative to glucose oxidation [3]. PDK has been widely studied in mammalian cells because of its clinical relevance. The inhibition of PDK alters the metabolism of cancer cells from glycolysis to glucose oxidation (GO) and reactivates mitochondria-dependent apoptosis [4]. In plants, PDK-repressed PDH activity has been suggested to modulate respiration and fatty acid biosynthesis [5–7]. Besides PDH, PDK is also capable of phosphorylating other proteins in eukaryotic cells [8]. Two PDKs have been found to modulate yeast growth by phosphorylating Pda1p, the *α*-subunit of PDH [9,10], but the study for the role of PDK in the regulation of fungal metabolism is still very limited.

The phytopathogen *Fusarium graminearum* Schwabe (teleomorph: *Gibberella zeae*) belonging to filamentous ascomycete causes Fusarium head blight (FHB) that is an economically important disease all over the world [11]. *F*. *graminearum* not only results in severe yield losses of crops by infecting cereals but also causes food safety incidents by producing mycotoxins [12]. However, *F*. *graminearum* populations can easily develop resistance to currently-used fungicides [13,14]. To develop the controlling strategy, it is important to deeply understand the regulatory mechanism for the growth, virulence, and toxin production of *F*. *graminearum*. PDK is considered as a novel therapeutic target in oncology [4]. To date, the physiological role of PDK in plant pathogens is still unclear. Therefore, it is of interest to investigate whether and how PDK regulates the intrinsic physiology of *F*. *graminearum*.

The aim of the present study was to identify the biological function of a putative PDK (FgPDK1) in *F*. *graminearum*. Genetic evidences supported the fact that FgPDK1 played vital role in the regulation of PDH activity, fungal development, stress responses, toxin production, and pathogenicity of *F*. *graminearum*. The possible mechanism of FgPDK1 driving these physiological processes, and their significance, were discussed as well.

## Methods

### Strains and culture conditions

Wild-type wheat-pathogenic *Fusarium graminearum* strain PH-1 was used as the wild-type strain for transformation in this study [15]. The strains were grown on potato glucose agar (PDA), complete medium (CM) [16], and minimal medium (MM) [17] for mycelial growth assays described in the figure legends. The mung bean broth (MBB) medium was used for sporulation assays [18]. The YEPD medium was used for conidial germination assays. The glucose yeast extract peptone (GYEP, 5% glucose, 0.1% yeast extract, and 0.1% peptone) was used for fungal culture prior to quantitative real-time RT-PCR (qRT-PCR) analysis for gene expression [19].

### Sequence analysis of *FgPDK1*

The sequence of PDK1 (also named as PKP1) from *Saccharomyces cerevisiae* was used as query for BLASTP search in the genome of *F*. *graminearum* (http://www.broadinstitute.org/annotation/genome/fusarium_group/MultiHome.html). to obtain the putative FgPDK1 (accession number FGSG_01963.3). The full-length genomic DNA and cDNA of *FgPDK1* were amplified, sequenced, and then compared with other fungi. The conserved domains and functional sites of FgPDK1 were analysed by using “Conserved domain database (CDD)” [20]. The multialignment of amino acid sequences was performed with ClustalX 2.0 and DNAMAN 5.2.2 [21]. Phylogenetic analysis was performed by the neighbour-joining method using MEGA v. 4.0.2 software [22].

### DNA and RNA extraction

Genomic DNA was isolated from mycelia using the optimized CTAB procedure [23]. RNA samples were isolated with the RNeasy kit (Tian gen, China) from 2-day-old mycelia grown in YEPD and GYEP liquid medium. First-strand cDNA was synthesized with the PrimeScript® RT reagent kit (TaKaRa). All qRT-PCR reactions were performed with an ABI 7500 real-time detection system (Applied Biosystems, USA). Primers used for qRT-PCR analysis are listed in S1 Table.

### Construction of the *FgPDK1* gene replacement vector

In order to analyse the function of the *FgPDK1* gene, an *FgPDK1* deletion mutant (ΔFgPDK1) was constructed in the PH-1 genetic background. The deletion vectors for *FgPDK1* were generated as described by our previous study [24]. First, the 1.0 kb upstream (1.0-up) and 1.0 kb downstream (1.0-down) of *FgPDK1* were amplified by using primer pairs A1+A2 and A3+A4 (S1 Table), respectively. The primers HTF/HTR were used to amplify a 1.5-kb fragment encoding the *HPH-tk* cassette containing the hygromycin resistance gene. This cassette was initially amplified from the PtrpChptA-PItk plasmid (data not shown) (S1 Table). Then, the double-joint PCR was applied to connect the amplified three fragments (1.0-up, 1.5-*hph*, and 1.0-down) [25]. Finally, the deletion vector was obtained by PCR amplification using the primer pair of P1+P4 by using the double-joint PCR products as the template. The PCR products were purified by using AxyPreTP DNA Gel Extraction Kit (Axygen Biosciences, China) according to the manufacturer’s instructions. The deletion vector was confirmed by restriction enzymes and sequencing.

### Transformation of *F*. *graminearum*

The transformation *F*. *graminearum* was performed according the previously published method [26]. The single-spore isolation were used to purify the putative transformants and were followed by the transfer to PDA plates with and without 100 μg/ml of hygromycin B. The deletion of *FgPDK1* was identified with the amplification using primer pair A5+A6 (S1 Table). Then primer pairs A7+R1 and F1+A8 (S1 Table) were used to amplify the fragment of the connecting area in order to confirm that *hph* was inserted into the original location of *FgPDK1* in the genome. The deletion mutants were further confirmed by using Southern blotting analysis. Basically, the genomic DNA of the transformants was digested with *SacI* and hybridized with probe1 (484 bp) by using primers P1+P2 (S1 Fig, S1 Table). Southern blotting was performed with the Dig High Primer DNA Labeling and Detection Starter Kit I (cat. no. 11745832910; Roche, Germany) were used to perform Southern blotting based on the manufacturer’s instructions.

### Complementation of *FgPDK1* deletion mutants

The *FgPDK1* deletion mutant (ΔFgPDK1) was complemented with the full-length of original *FgPDK1* to confirm the phenotypic changes of *FgPDK1* deletion mutants. The original *FgPDK1* gene amplified with primers A9/ A10 (S1 Table) was inserted into pYF11 (bleomycin resistance) vector. Then the construct was used for protoplast transformation of the ΔFgPDK1 mutant. The obtrained transformants were first screened by phenotypic characterization, followed by PCR amplification, and verified by fully restored growth.

### Test of sensitivity to compounds causing osmotic stress, cell wall stress, and cytoplasm membrane stress

Mycelial growth tests with strains PH-1, ΔFgPDK1, and ΔFgPDK1-C were performed on PDA plates supplemented with the NaCl, KCl, caffeine, congo red, and SDS (sodium dodecyl sulfonate), respectively. Each plate was inoculated with a 5-mm-diameter mycelial plug taken from the edge of a 3-day-old colony. The percentage of mycelial radial growth inhibition (RGI) was calculated by using formula (1).

RGI=((A−B)/(A−5))×100 (1)

*A* and *B* indicate the colony diameter of the control treatment, respectively. Each experiment was independently repeated three times [27].

### Test of conidiation and perithecium production

For a conidiation assay with strains PH-1, ΔFgPDK1, and ΔFgPDK1-C, five mycelial plugs (5 mm diameter) obtained from the edge of a 3-day-old colony were cultured in a flask containing 100 ml of MBB (170 rpm, 25°C, with light) [28]. The spores were counted with a hemacytometer after 7 days. Each strain was performed by three replicate flasks, and the experiment was repeated three times independently.

For a perithecium production assay for all the tested strains, ten mycelial plugs (5 mm diameter) obtained from the edge of a 3-day-old colony were transferred to sterile 50-mL flasks containing moist and autoclaved wheat seeds (cultivar Yangmai no. 49, about 30 seeds per flask). After maintain at 25°C for 7 days, the seeds were transferred to 9-cm-diameter Petri plates covered with sterile wet sand, and placed in a humid room (RH 80%, 25°C, 12-h photoperiod). After another 15 days, the seeds were scored as ‘+++’, ‘++’, or ‘+’ when perithecia covered more than 2/3, between 1/3 and 2/3, or less than 1/3 of each seed, respectively. Each strain was performed by three replicate flasks, and the experiment was repeated twice independently.

### Determination of relative conductivity and lipid peroxidation

The relative conductivity of strains PH-1, ΔFgPDK1, and ΔFgPDK1-C were measured according to the method described previously [29]. Mycelial plugs (5 mm diameter) from the margins of 3-day-old colonies on PDA were placed in 250 mL flasks (5 plugs per flask) containing 100 mL of PDB. The flasks were placed on a rotary shaker (175 rpm at 25°C). After 48 h, mycelia were collected on double gauze and washed twice with double-distilled water. After filtration in vacuum for 20 min, 0.5 g of mycelia per sample was suspended in 20 mL of double-distilled water. The electrical conductivity of the double-distilled water was measured with a conductivity meter (CON510 Eutech/Oakton, Singapore) at 0, 10, 20, 40, 60, 80, 100, 120, 140, 160 and 180 min, respectively, to assess the extent of leaching of cell contents through cell membranes. After 180 min, the mycelia were boiled for 5 min for the measurement of final conductivity. The relative conductivity of mycelia was calculated with formula (2).

Relativeconductivity(%)=Conductivity×100/Finalconductivity (2)

The concentration of malondialdehyde (MDA) was determined as an indicator of the level of lipid peroxidation in *F*. *graminearum*. A MDA detection kit (A003; Nanjing Jiancheng Bioengineering Institute, Nanjing, China) was selected to determine the MDA level based on the spectrophotometric determination of the reaction between MDA and 1,3-diethyl-2-thiobarbituric acid (TBA) assisted by trichloroacetic acid (TCA) [30].

### Histochemical detection of cell membrane permeability and ROS (reactive oxygen species) accumulation

The cell membrane permeability was detected with fluorescent probe PI (propidium iodide) that can only enter membrane-compromised cells [31]. Fresh mycelia were harvested and incubated in 2 μM of PI for 20 min, and rinsed with distilled water for three times followed by the visualization with a fluorescence microscope (excitation 535 nm and emission 615 nm) (ECLIPSE, TE2000-S, Nikon).

Intracellular ROS was visualized using fluorescent probe DCFH-DA (2′,7′-dichlorofluorescein diacetate) described in our previous study [32]. Fresh mycelia were harvested and incubated in 1 μM of DCFH-DA for 20 min, and rinsed with distilled water for three times followed by the visualization with a fluorescent microscope (excitation 488 nm and emission 525 nm) (ECLIPSE, TE2000-S, Nikon).

### Determination of PDH activity

A PDH detection kit (MAK183; SIGMA-ALDRICH) was selected to determine the PDH activity assay was based on NADH production. PDH activity is determined using a coupled enzyme reaction, which results in a colorimetric (450 nm) product proportional to the enzymatic activity present. One unit of pyruvate dehydrogenase is the amount of enzyme that will generate 1 μmole of NADH per minute at 37°C.

### Analysis of DON production and expression levels of *Tri5* and *Tri6*

For the quantification of DON production *in vitro*, conidia of strains PH-1, ΔFgPDK1, and ΔFgPDK1-C were collected and suspended in GYEP liquid medium at a concentration of 1×10^5^ conidia/ml. The young mycelium was filtered, dried, and weighed after 7 days at 28°C in the dark. The volume of collected filtrate was recorded. Then 20 ml of filtrate was extracted with 20 ml of ethyl acetate and was distilled to obtain a DON sample The DON sample was re-suspended in 100 ml of TMSI: TMCS (100:1) followed by incubation at 37°C for 1 h. After that, 1 ml of isooctane and 800 μl of sterile water were added to the mixture. After the aqueous and organic layers were completely separated, the top phase was analysed by using GC-ECD (gas chromatography-election capture detector). Nitrogen was used as the carrier gas at a constant flow rate of 1 ml/min through an Agilent HP-5 (30 m × 320 μm × 0.25 μm) capillary column with an injector temperature of 300°C. DON production was represented as per mg of mycelium.

For the quantification of the expression levels of two trichothecene biosynthesis genes (*Tri5* and *Tri6*), mycelia of each strain were added to GYEP liquid medium and cultured at 28°C for 2 days in the dark. Total RNA was extracted from the mycelia of each sample by using Trizol (Invitrogen) according to the manufacturer’s instructions. The expression levels of *Tri5* and *Tri6* were determined with real-time quantitative reverse transcription-polymerase chain reaction (qRT-PCR) (Applied Biosystems 7500 Fast Real-Time PCR System, LifeTechnologies). The primer pairs (P17+P18) and (P19+P20) were used for the amplification of *Tri5* and *Tri6*, respectively (S1 Table).

### Test of pathogenicity on flowering wheat heads and fresh tomatoes

Wheat plants (cultivar Yangmai no. 49) grown from seed were used to test the virulence of strains PH-1, ΔFgPDK1, and ΔFgPDK1-C as described previously [33]. One mycelial plug (about 2 mm^2^) of each strain taken from the edge of a 4-day-old colony was injected to the two outer florets of the centre spikelet, then 10 μl of distilled water was supplied to maintain moisture. Wheat spikes injected with one medium plug and distilled water were used as control group. Thirty replicated heads were prepared for each tested strain. Disease severity was calculated as the percentage of blighted spikelets in each head after inoculation for 16 days.

Fresh tomatoes were inoculated with mycelia as previously described [34]. After 5 days at 30°C with 12 h of light per day, the lesions were measured. Each mutant or strain was represented by three replicate fruits, and the experiment was performed three times independently.

### Statistical analysis

Data shown are mean standard deviation (SD) of at least three replicated measurements. Significant differences between treatments were statistically evaluated by SD and one-way analysis of variance (ANOVA) using SPSS 2.0. Two specific different treatments were compared statistically by ANOVA, followed by F-test if the ANOVA result was significant at *P*<0.05. For multiple comparison analyses, least significant difference test (LSD) was performed on all data after ANOVA when significant differences (*P*<0.05) were detected among different treatments.

## Results

### Characterization of the *FgPDK1* gene

**Fig 1:**
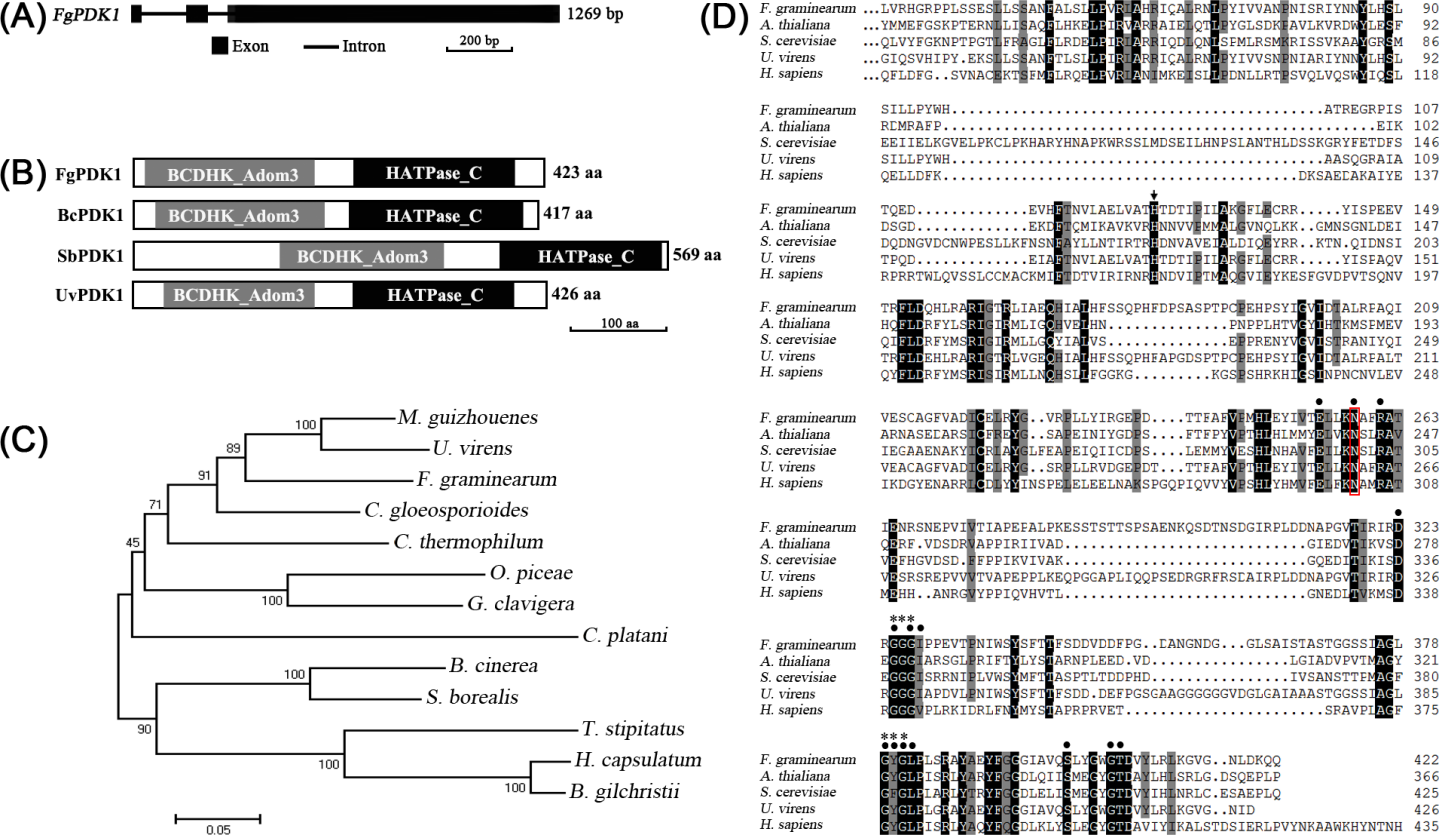
Sequence analysis of FgPDK1. (A) Structure of nucleic acid sequence of *FgPDK1*. (B) Analysis of conserved domains in FgPDK1 and its homologues from other fungi. (C) The phylogenetic analysis of PDK1s from different fungi. The phylogenetic tree was constructed using the Neighbour-Joining method. Evolutionary analyses were conducted in MEGA4. (D) The multialignment of PDK1 from *F*. *graminearum*, *A*. *thialiana*, *S*. *cerevisiae*, *U*. *virens*, and *H*. *spiens*. *Dark shading* with *white letters* and *grey shading* with *black letters* indicate 100% and 75% sequence similarity, respectively. The *black dot* indicates ATP binding site. The *asterisk* indicates G-X-G motif. The *red box* indicates Mg^2+^ binding site. The *black arrow* indicates conserved His working for phosphotransfer.

The *FgPDK1* gene (accession number FGSG_01963.3) was retrieved from *F*. *graminearum* genome sequence deposited in the Broad Institute (http://www.broadinstitute.org) by using BLAST. The *FgPDK1* gene is interrupted by two introns. The first intron is 159 bp long and is located between the 34th and 194th nucleotides; the second intron is 59 bp long and is located between the 257th and 317th nucleotides (Fig 1A). It has an open reading frame (ORF) that encodes 423 amino acids containing two typical PDK domains: a BCDHK (mitochondrial branched-chain alpha-ketoacid dehydrogenase kinase) domain (24 aa-202 aa) and a HATPase (hisdine kinase-like ATPase) domain (248 aa-412 aa) (Fig 1B). FgPDK1 shares a high similarity with other fungal PDK proteins, such as *Ustilaginoidea virens* GAO15741.1, *Sclerotinia borealis* ESZ97745.1, and *Botrytis cinerea* EMR87909.1 (Fig 1C). The multialignment suggested that PDKs from different species contained all the conserved functional sites, such as ATP binding sites, Mg^2+^ binding sites, and G-X-G motif (Fig 1D). In *Arabidopsis thialiana*, the His-121 residue located in BCDHK domain is essential for phosphotransfer during PDK-conducted phosphorylation activity [6]. Here we also found the His-126 in FgPDK1, and this residue is conserved in the PDKs from human, yeast, *A*. *thialiana*, and *U*. *virens* (Fig 1D).

### Targeted knockout of the *FgPDK1* gene and complementation in *F*. *graminearum*

To examine the cellular function of *FgPDK1*, a gene replacement construct (S1A Fig) was made by ligation PCR and the ΔFgPDK1 mutant allele was used to transform PH-1 to obtain the ΔFgPDK1 mutant(s). The expected size of the whole transformation fragment was 3785 bp. The primer pairs P7+R1 and F1+P8 amplified 2,181-bp and 2,301-bp fragments from the *FgPDK1* deletion mutant ΔFgPDK1 but did not amplify any fragments from the wild-type strain PH-1 (S1B Fig). In southern blot hybridization analyses, the deletion mutant ΔFgPDK1, but not wild-type PH-1, produced an anticipated 2,879-bp band detected with probe1 (S1C Fig). The ΔFgPDK1 mutant was complemented with the parental gene *FgPDK1*. The putative complementation was examined by Southern blot analysis as well (S1C Fig).

### *FgPDK1* is essential for the repression of PDH activity and morphogenesis of *F*. *graminearum*

**Fig 2:**
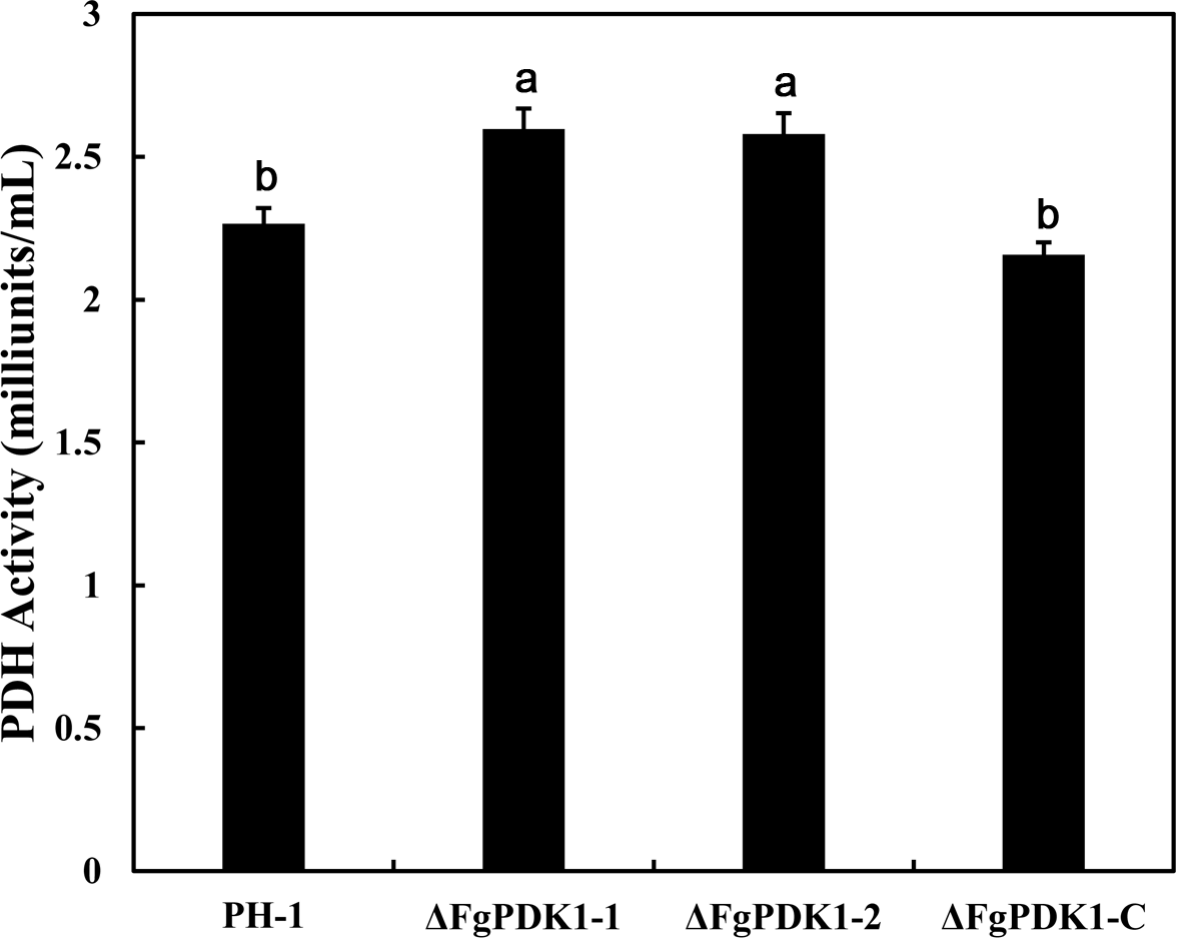
Effect of *FgPDK1* deletion on PDH activity in *F*. *graminearum*. The PDH activity was determined in mycelia of wild-type strain PH-1, the *FgPDK1* deletion mutant (ΔFgPDK1), and the complemented strain (ΔFgPDK1-C), respectively. The mean values of three replicates followed by different letters indicated significance of difference between the treatments (*P*<0.05, ANOVA, LSD).

To address the question of whether the FgPDK1 is an essential prerequisite for the regulation of mitochondrial PDH activity, the mitochondria of the null mutant strains were isolated and tested for PDH activity by monitoring the production of NADH in the presence of pyruvate as a substrate. As expect, the deletion of *FgPDK1* resulted in the significant increase in the activity of mitochondrial PDH compared with wild-type and the complemented strain (Fig 2).

**Fig 3:**
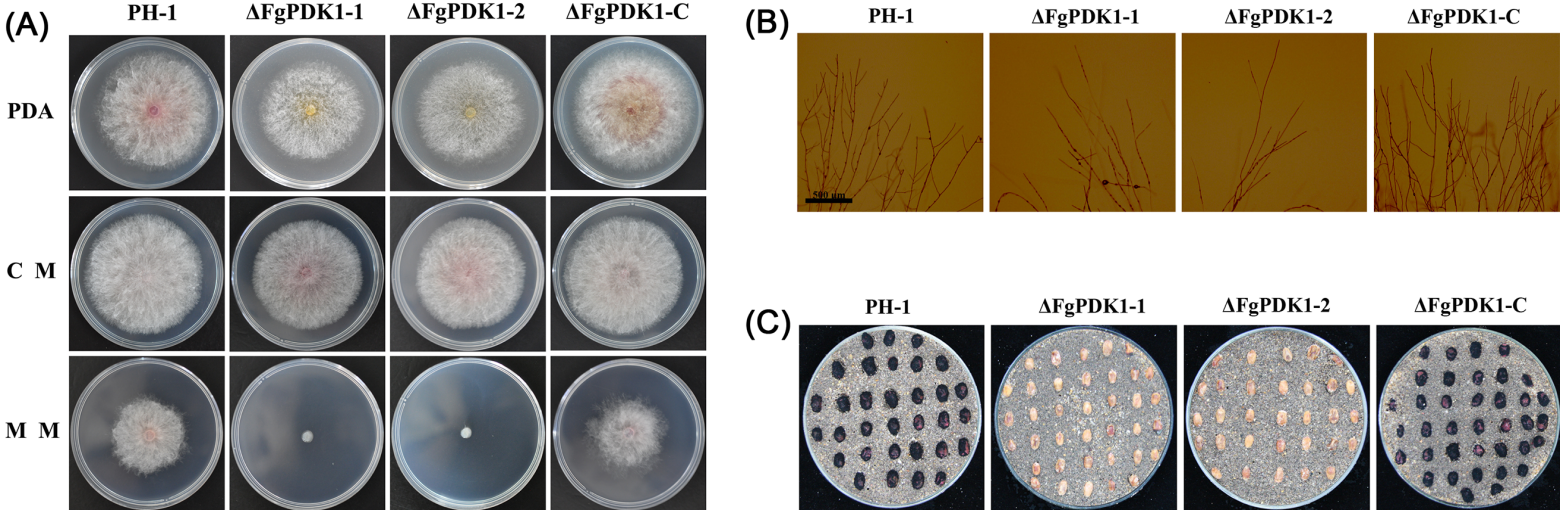
Effect of *FgPDK1* deletion on *F*. *graminearum* colony morphology and perithecia production. (A) The wild-type strain (PH-1), *FgPDK1* deletion mutant (ΔFgPDK1), and complemented strain (ΔFgPDK1-C) were grown on PDA, CM, and MM for 4 days at 25°C. (B) Micrographs of hyphae of PH-1, ΔFgPDK1, and ΔFgPDK1-C. (C) Black perithecia production on autoclaved wheat seeds.

The growth of the ΔFgPDK1 mutants was evaluated on PDA, CM, and MM plates. The ΔFgPDK1 mutants showed different growth patterns as compared with that of the wild-type and the complemented mutant strain (ΔFgPDK1-C) (Fig 3A and Table 1). ΔFgPDK1 grew slightly slower than the wild-type parent and the hyphae turned yellow and white on PDA medium. Expression of the yellow pigment-formation genes *AurO* and *AurR2* were significantly up-regulated in the deletion mutants while the expression of red pigment-formation genes *AurJ* and *AurF* were not changed significantly (S2 Fig). It is interesting to notice that the deletion mutant grew significantly slower on MM medium with limited carbon source (only sucrose) while it grew normal on CM medium (Table 1 and Fig 3A). Microscopic examination showed that the deletion of FgPDK1 did not change the morphology of aerial hyphae compared with the wild-type parent (Fig 3B). Perithecium can release ascospore (S3 Fig), which is responsible for the sexual reproduction of *F*. *graminearum*. Compared to wild-type strain and the complemented strain, the ΔFgPDK1 mutant failed to produce any perithecia on autoclaved wheat seeds (Fig 3C). Conidium is important for *F*. *graminearum* to finish asexual reproduction. In the present study, after growing in MBB medium for 7 days, the ΔFgPDK1 mutant failed to produce any conidia (Table 1). All of these growth characteristics were restored in the complemented mutant ΔFgPDK1-C. These results suggested that the deletion of FgPDK1 remarkably impacted both sexual and asexual development of *F*. *graminearum*.

**Table 1:** Biological properties of the *F*. *graminearum* deletion mutant ΔFgPDK1, the complemented strain ΔFgPDK1-C, and the wild-type strain PH-1.

Strain	Growth rate in vitro (mm/day)			Conidia produced *in vitro* (×10^5^/ml)	Perithecia production	Pathogenicity on wheat spike (%)
	PDA	CM	MM			
**PH-1**	20±0.29a	21.33±0.29a	13.77±0.62a	1.58±0.39a	+++	57.3±11.3a
**ΔFgPDK1-1**	7.33±0.29b	3.11±0.45b	3.44±0.45b	0b	–	6±1.22b
**ΔFgPDK1-2**	7.22±0.45b	3.22±0.62b	3.89±0.45b	0b	–	7.6±2.18b
**ΔFgPDK1-C**	20.22±0.45a	20.89±0.17a	13.11±0.46a	2.13±0.45a	+++	56.7±12.9a

“+++” indicates that perithecia cover more than 2/3 of the wheat seed surface. “–” indicates that perithecia are not found on the surface of wheat seed. Values are the means±standard error of three replicates. Means in a column followed by the same letter are not significantly different according to the LSD test at *P*<0.05. The perithecia production was tested on autoclaved wheat seeds. The pathogenicity of strains on wheat spike was detected as the percentage of blighted spikelets per wheat head 16 days after inoculation of flowering.

### The stress responses of ΔFgPDK1 deletion mutant

**Fig 4:**
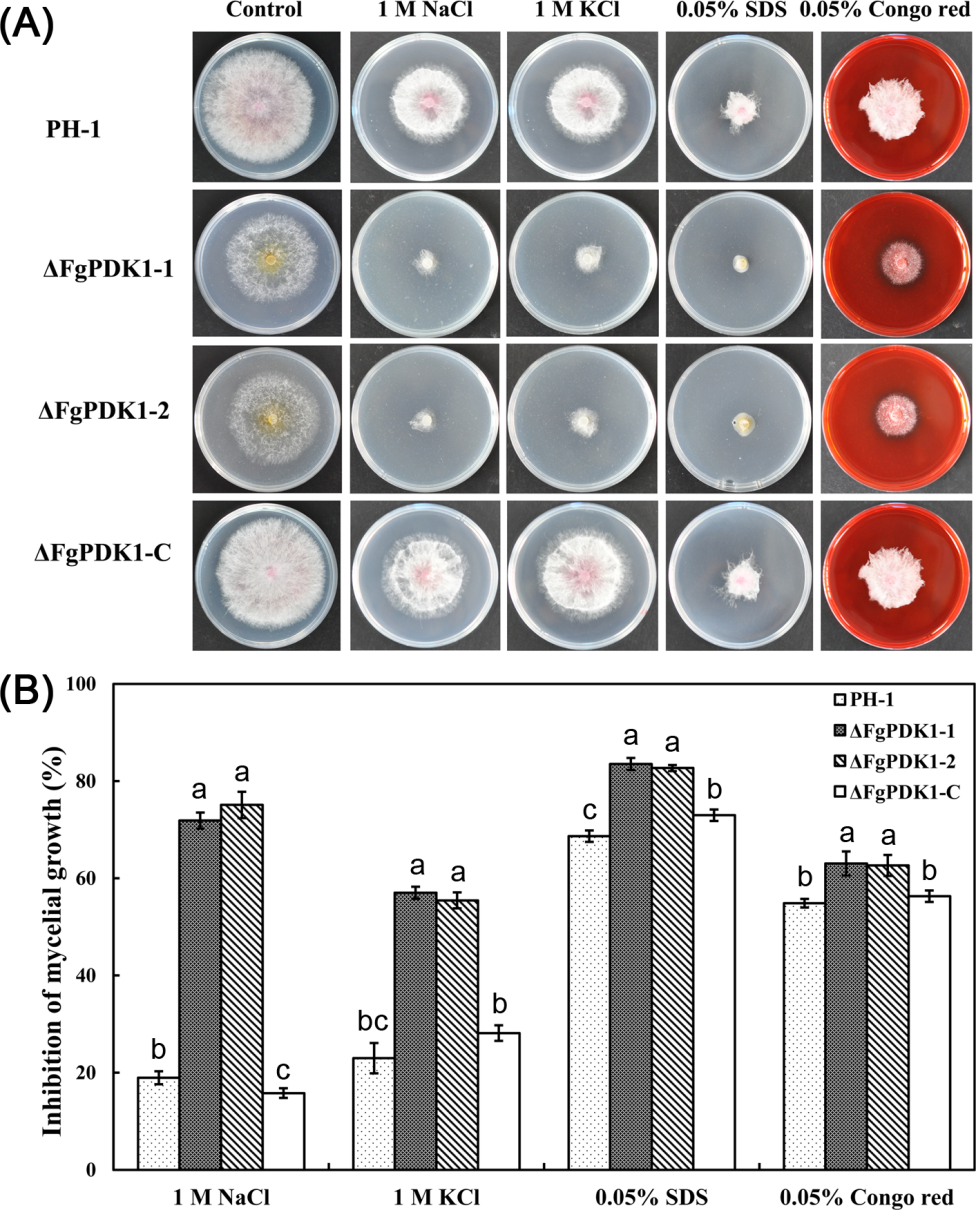
Stress response of the wild-type strain PH-1, the *FgPDK1* deletion mutant (ΔFgPDK1), and the complemented strain (ΔFgPDK1-C). The strains were grown in medium containing different chemicals as indicated. Then the photographs were taken (A), and the inhibition of mycelial growth were measured (B). The mean values of three replicates followed by different letters indicated significance of difference between the treatments (*P*<0.05, ANOVA, LSD).

In order to ascertain the possible role of FgPDK1 in stress responses of *F*. *graminearum*, the ΔFgPDK1 mutants were exposed to several stress-induced reagents (Fig 4). The growth of ΔFgPDK1 mutants was inhibited pronouncedly compared to wild-type strain and the complemented strain under the exposure of NaCl and KCl (Fig 4A), suggesting that the deletion of *FgPDK1* increased the sensitivity of *F*. *graminearum* to osmotic stress. Treatment with SDS, an inducer of cell membrane damage, resulted in the significant increase in the inhibition rate of mycelial growth of ΔFgPDK1 mutants than that of wild-type and the complemented strain (Fig 4B). However, there were no remarkable changes in mycelial growth among all the tested strains under the exposure of Congo red that can destroy cell wall integrity (Fig 4B). These results suggested that FgPDK1 may be an important regulator of cell membrane in stress responses of *F*. *graminearum*.

### Involvement of *FgPDK1* in the regulation of cell membrane permeability

**Fig 5:**
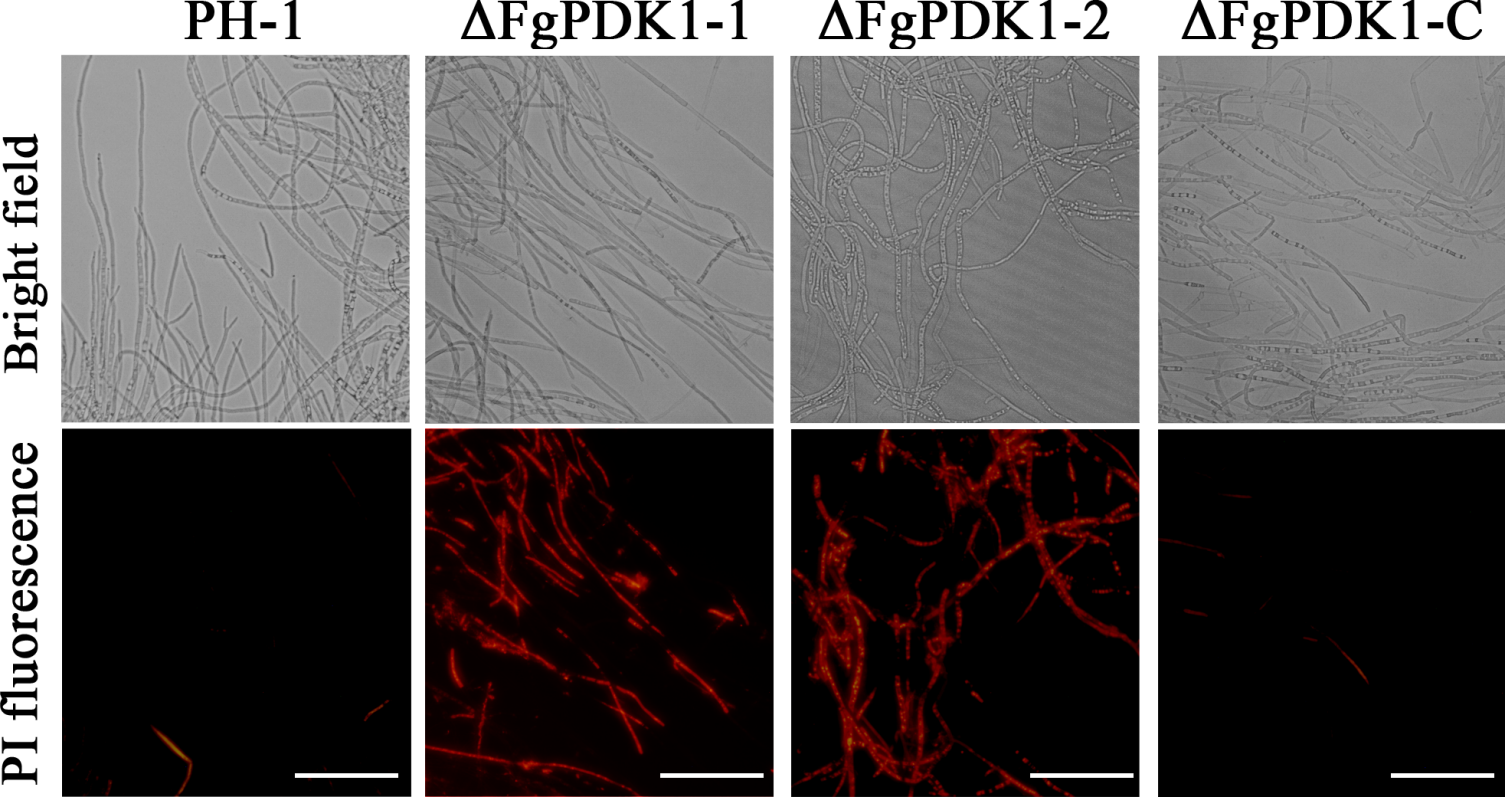
The fluorescent staining of membrane permeability in the mycelia of PH-1, the *FgPDK1* deletion mutant (ΔFgPDK1), and the complemented strain (ΔFgPDK1-C). Mycelia were incubated with PI followed by the fluorescent microscopic observation.

**Fig 6:**
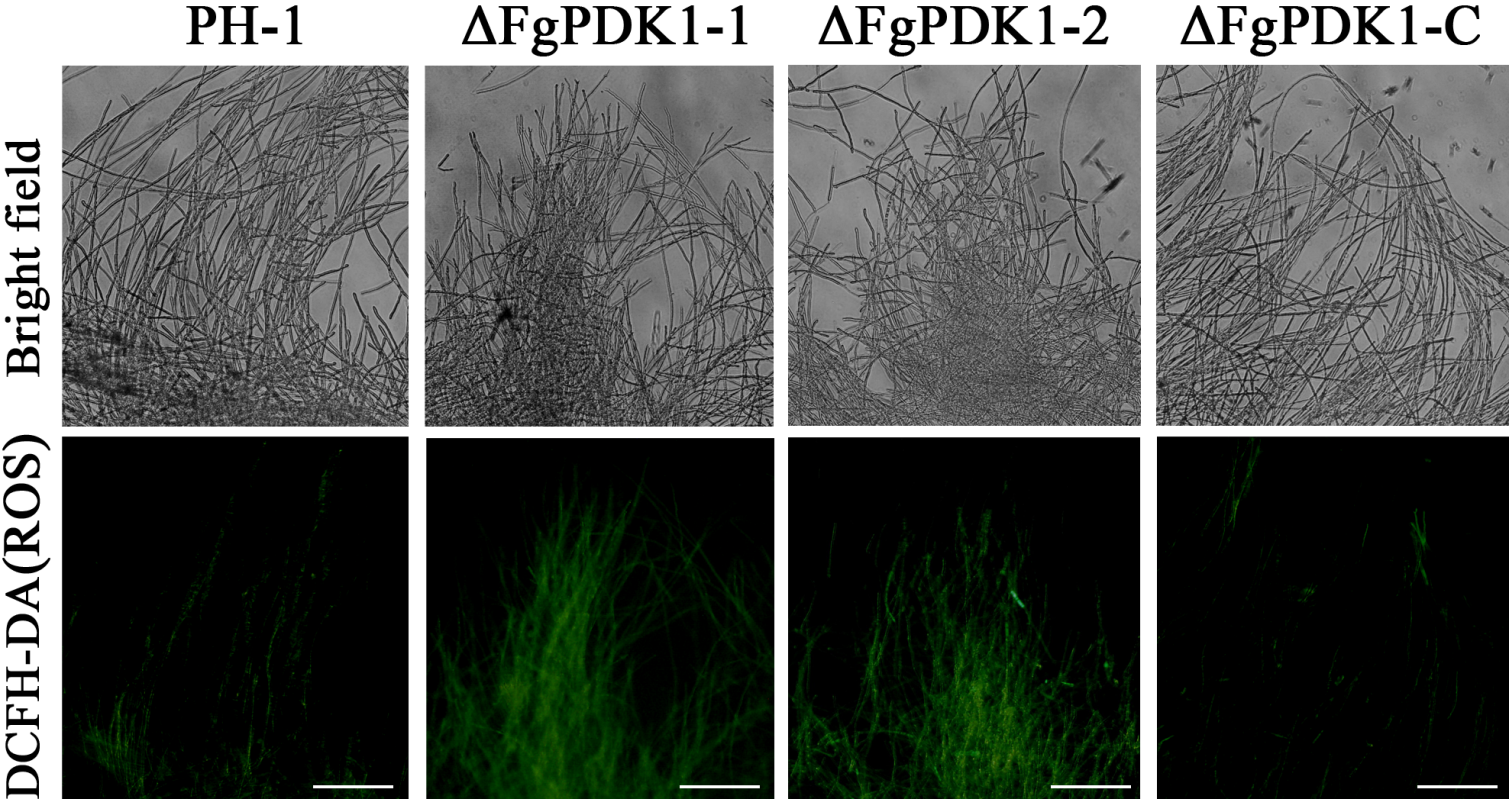
The fluorescent staining of ROS in the mycelia of PH-1, the *FgPDK1* deletion mutant (ΔFgPDK1), and the complemented strain (ΔFgPDK1-C). Mycelia were incubated with DCFH-DA followed by the fluorescent microscopic observation.

To test the cell membrane integrity, we performed PI staining that can only enter membrane-compromised cells to bind nucleic acids [31]. The PI-stained hyphae of ΔFgPDK1 mutants showed more extensive red fluorescence than that of wild-type and the complemented strain (Fig 5). As an important factor for damaging cell membrane, the endogenous ROS detected by specific probe DCFH-DA increased in ΔFgPDK1 mutants than that of wild-type and the complemented strain (Fig 6).

**Fig 7:**
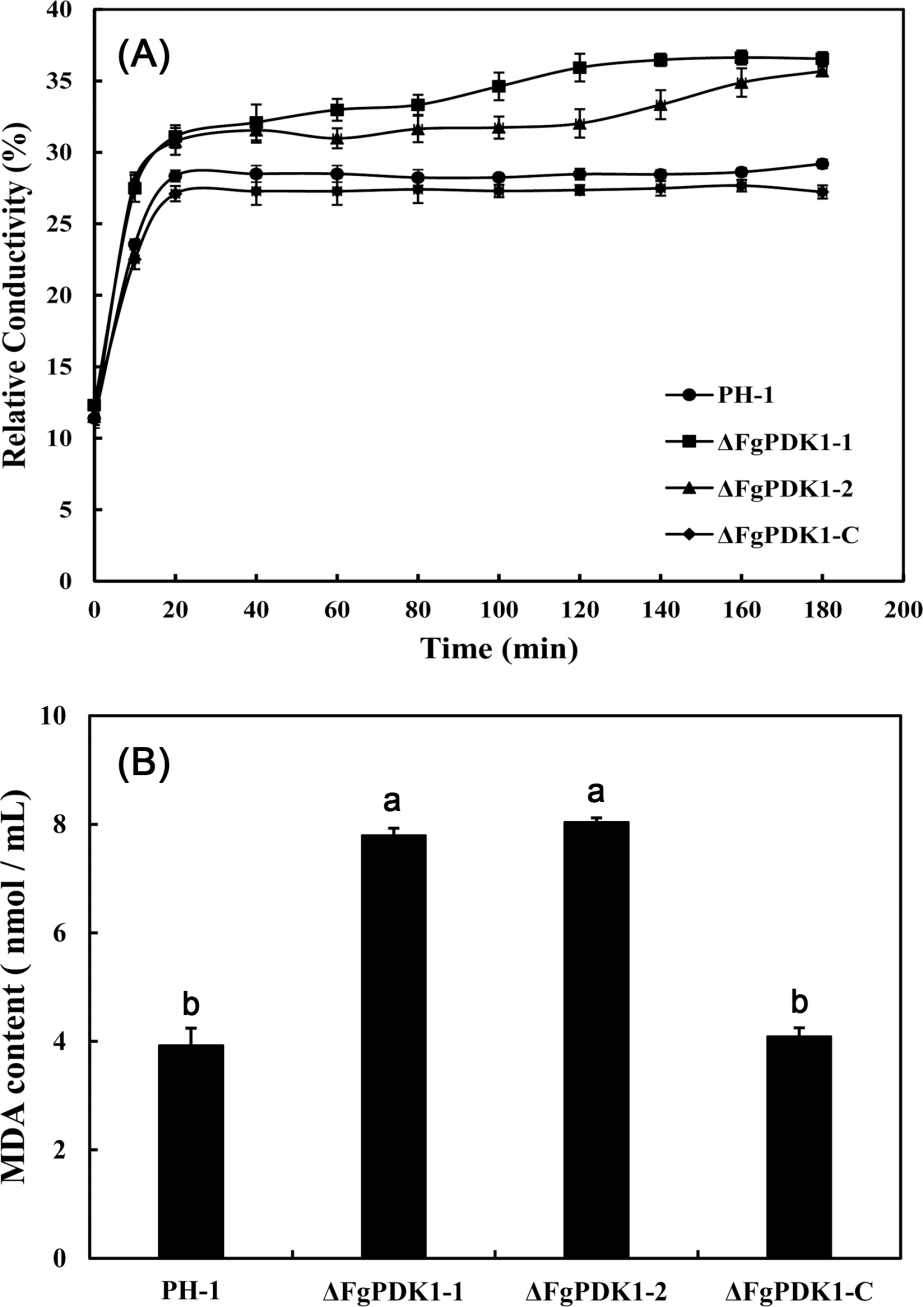
**Relative conductivity (a) and MDA content (b) of mycelia in PH-1, the *FgPDK1* deletion mutant (ΔFgPDK1), and the complemented strain (ΔFgPDK1-C).** The mean values of three replicates followed by different letters indicated significance of difference between the treatments (*P*<0.05, ANOVA, LSD).

The increase in cell membrane permeability results in the increase in electrolyte leakage, which can be indicated by relative conductivity. In a time-course experiment, the relative conductivities obtained from the distilled water containing mycelia of ΔFgPDK1 mutants were higher than that of wild-type progenitor and the complemented strain (Fig 7A). MDA concentration has been frequently used as an index of lipid peroxidation, indicating the oxidative injury of cell plasma membrane [35]. Compared to wild-type and the complemented strain, the deletion of *FgPDK1* led to the significant increase in MDA content in mycelia (Fig 7B). These results suggested that FgPDK1 was important for maintaining cell membrane integrity of *F*. *graminearum*.

### Involvement of FgPDK1 in the regulation of DON biosynthesis

**Fig 8:**
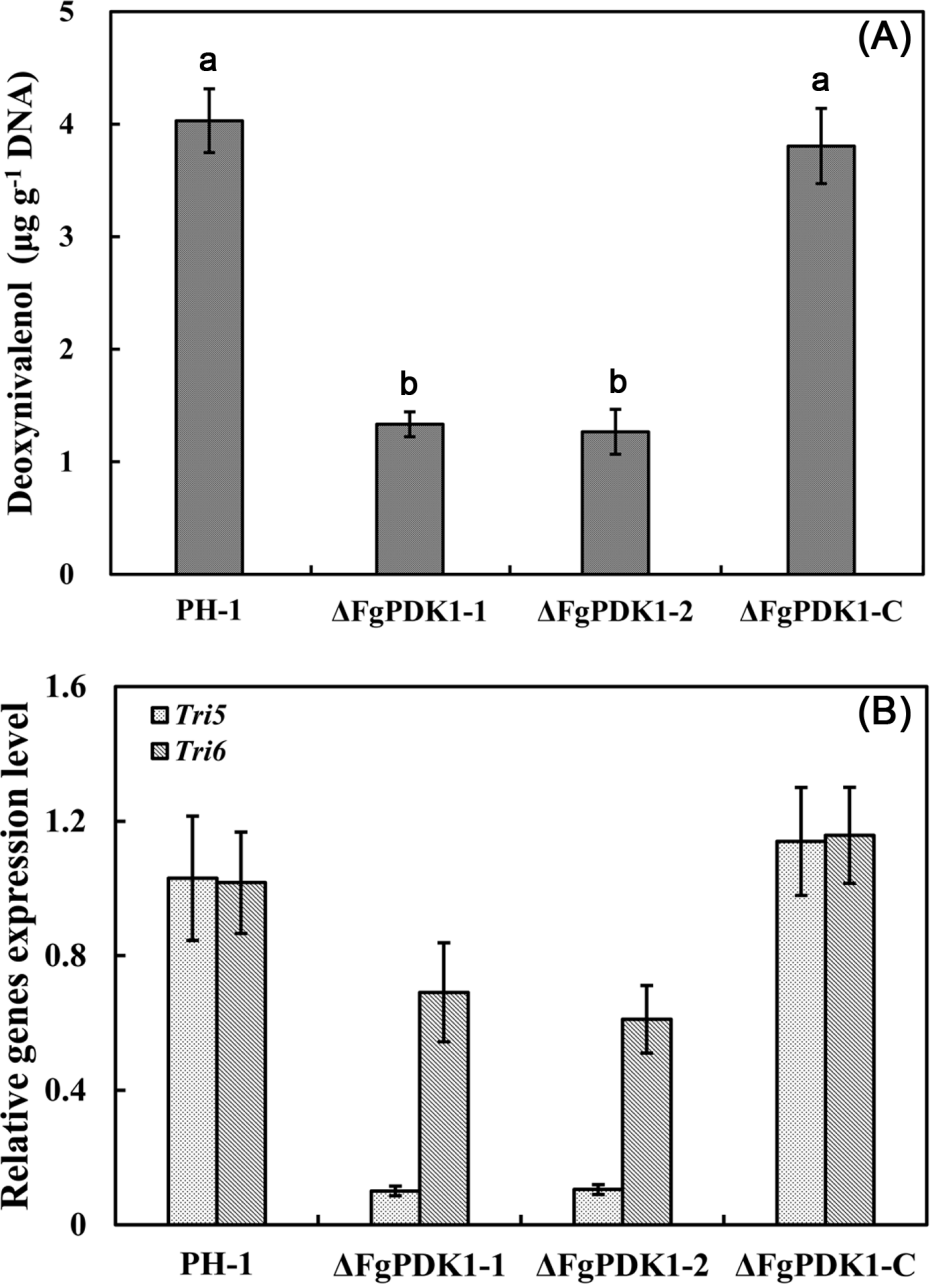
**Deoxynivalenol (DON) production (A) and relative expression of *Tri5* and *Tri6* (B) in PH-1, the *FgPDK1* deletion mutant (ΔFgPDK1), and the complemented strain (ΔFgPDK1-C).** The mean values of three replicates followed by different letters indicated significance of difference between the treatments (*P*<0.05, ANOVA, LSD).

DON (deoxynivalenol), belonging to trichothecene family, is one of most frequently studied mycotoxins produced by *F*. *graminearum*. DON not only poses threat to food safety but also acts as a probable virulence factor that helps the fungus establish and spread within spikes [36,37]. In the present study, the wild-type and the complemented strain produced three-times more DON than ΔFgPDK1 mutant (Fig 8A). Additionally, we investigated the expression levels of two trichothecene biosynthesis genes *Tri5* and *Tri6* by quantitative real-time PCR using RNA extracted from mycelia grown in GYEP medium. The expression levels of *Tri5* and *Tri6* were significantly lower in ΔFgPDK1 mutant than in wild-type and complemented strain (Fig 8B). These data indicated that FgPDK1 played an important role in the regulation of DON biosynthesis in *F*. *graminearum*.

### Involvement of FgPDK1 in the regulation of *F*. *graminearum* pathogenicity

**Fig 9:**
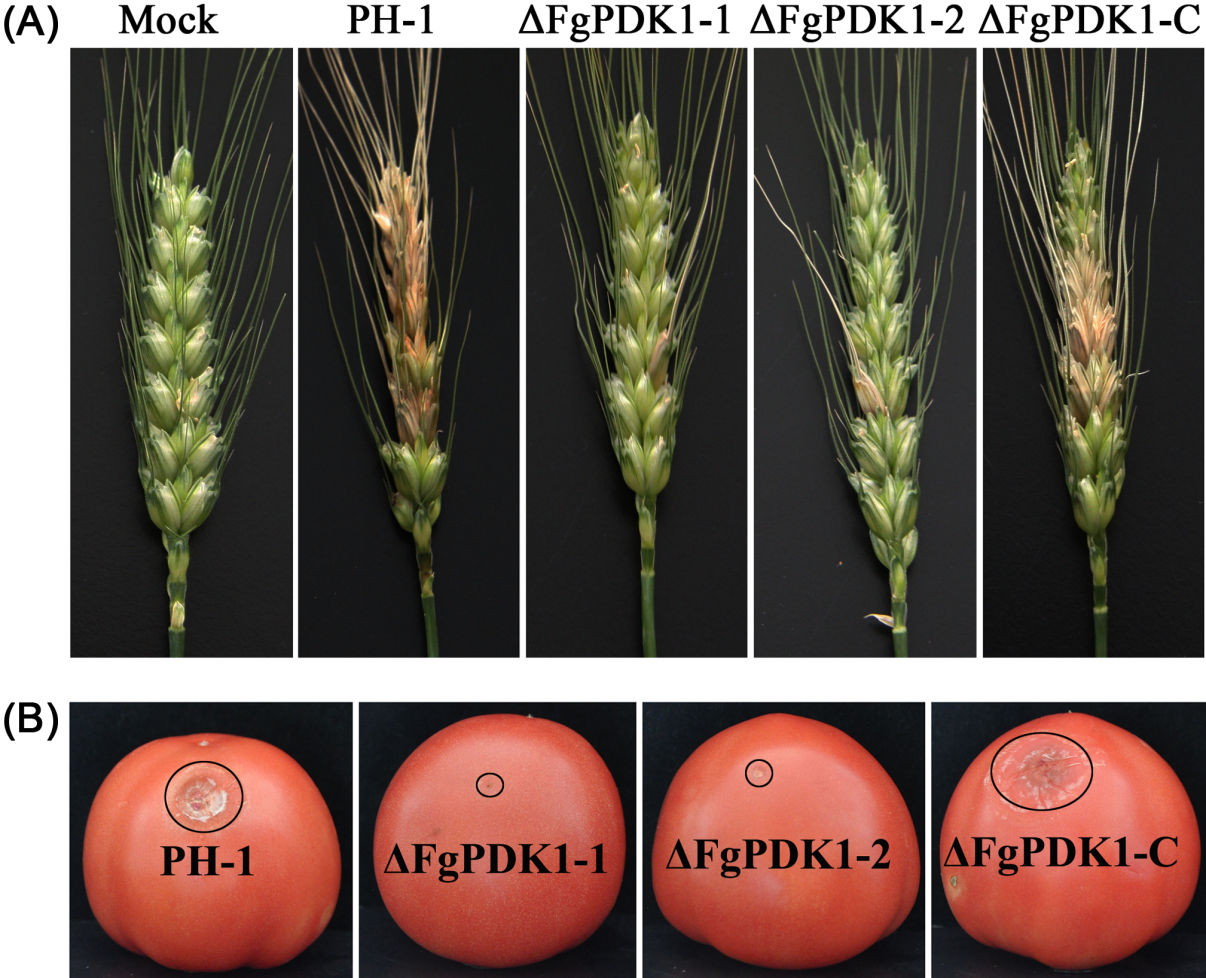
Pathogenicity assays for strain PH-1, the *FgPDK1* deletion mutant (ΔFgPDK1), and the complemented strain (ΔFgPDK1-C). (A) Symptoms on flowering wheat heads inoculated with water (mock) or with mycelium of strains. Wheat heads were photographed 2 weeks after inoculation. (B) Lesions caused by the infection of different strains as indicated. Lesions were photographed after 5 days at 100% RH at 30°C.

Both wheat spikes and tomatoes were selected to test the pathogenicity of ΔFgPDK1 mutants. Fifteen days after flowering wheat heads were point inoculated with mycelial plug, the wild-type and the complemented strain induced scab symptoms on >50% of the inoculated wheat head while ΔFgPDK1 had reduced symptoms only near the point of inoculation (Fig 9A and Table 1). The similar results were also obtained from tomatoes, which wild-type and ΔFgPDK1-C produced serious lesions while ΔFgPDK1 mutants failed to infect tomatoes (Fig 9B). All these results demonstrated that the full virulence of *F*. *graminearum* required FgPDK1.

## Discussion

PDK is an important regulator of PDC that is related to two major energy-generating pathways, glycolysis and TCA cycle [38]. The pivotal role of PDK in metabolic flexibility has been reported in mammals, plants, and yeast [10,38,39], but its biological function in plant pathogens remains elusive. Here we provide the evidences to suggest that FgPDK1 is a multifunctional modulator in an economically important phytopathogen *F*. *graminearum*.

PDK is a gate-keeping mitochondrial enzyme that regulates the flux of carbohydrates via pyruvate from the cytoplasm into the mitochondria, where glucose oxidation occurs. In mammalian cells, the activation of PDH by the down-regulation of PDK can switch fatty acid utilization to glucose catabolism [38]. This may explain the observation that the deletion of *FgPDK1* mutant with enhanced activity of mitochondrial PDH showed decreased carbon utilization capacity on MM containing solo carbon source (sucrose). The similar phenotype has been also reported in yeast, which the disruption of PDK1 resulted in the retarded growth on glucose-free media [10]. Pyruvate is an important metabolic regulator connecting glycolysis-gluconeogenesis, TCA cycle, and lipid metabolism. Besides PDH, pyruvate also serves as substrate for other carbon metabolic-related enzymes during fungal growth, such as pyruvate decarboxylase, lactate dehydrogenase, pyruvate carboxylase, etc. In the present study, the enhanced PDH activity resulted from the deletion of *FgPDK1* may impact the utilization of pyruvate by other pathways (e.g. lipid synthesis). One of the evidences from mammalian study is that inactivation of PDH by PDKs promotes the shift of pyruvate to fatty acid synthesis in liver cells under the condition of energy deprivation [38,40]. Glycolysis and gluconeogenesis, two reversible metabolic processes, are fine-tuned in filamentous fungi *Aspergillus nidulans*. *A*. *nidulans* can utilize carbon sources metabolized from TCA cycle requiring gluconeogenesis [41,42]. In our present study, the deletion of *FgPDK1* may disrupt the balance between glycolysis and gluconeogenesis, which in turn results in the deprivation of energy generated from TCA cycle.

In the present study, the increased MDA content and relative conductivity in ΔFgPDK1 mutants suggested that FgPDK1 was important for maintaining cell plasma membrane integrity and stability. This may explained the increased sensitivity of ΔFgPDK1 mutants in response to osmotic stress induced by salt or SDS. Stress conditions frequently induce the over-generation of ROS, which further cause cell membrane injury through lipid peroxidation [43,44]. The PDK1 knockout yeast mutant showed high sensitivity to hydrogen peroxide-induced oxidative stress [45], which is similar to our current observation that the deletion of FgPDK1 led to the accumulation of ROS in *F*. *graminearum*. Therefore, it can be concluded that FgPDK1 may protect cell membrane from stress by decreasing ROS. The capability of PDK1 to scavenge ROS may be attributed to lower mitochondrial respiration [46].

The disturbance of plasma membrane potential is one of the major consequences of osmotic stress. The plasma membrane H^+^-ATPase is an essential enzyme for the maintenance of membrane potential in osmotic tolerance [47]. Interestingly, the screening of yeast membrane potential phenotype of protein kinase mutants suggested that PDK1 might be directly related to the post-translational modification of H^+^-ATPase through phosphorylation in yeast cells [8]. In *F*. *graminearum*, whether FgPDK1 regulates membrane potential through the similar mechanism remains to be studied further.

DON biosynthetic enzymes and direct regulatory proteins are encoded by *Tri* genes [48]. Among them, *Tri6* has been considered as an importantly regulatory node during the biosynthesis of DON. *Tri6* encodes a zine finger transcription factor capable of enhancing the expression of various *Tri* genes, including *Tri5* encoding the trichodiene synthase that catalyses the first biosynthetic step of DON [48–50]. In the present study, the deletion of FgPDK1 dampened the production of DON, which may result from the decrease in the expression of *Tri5* and *Tri6*. In addition, we found that the pathogenicity of ΔFgPDK1 mutant was weakened on wheat spikes, and the mutant almost lost pathogenicity on tomatoes. It has been reported that the *Tri6* deletion mutant of *F*. *graminearum* shows greatly reduced pathogenicity [51]. Thus, *Tri6* may act downstream of FgPDK1 for the full virulence of *F*. *graminearum*. Besides, *Tri6* is a global transcription regulator in the overall growth and development of *F*. *graminearum* [52]. Therefore, the repression of *Tri6* expression may be directly linked to the all the phenotype defect of ΔFgPDK1 mutant observed in this study. Given the upstream of Tri6 is poorly understood, the mechanism for the regulation of *Tri6* by FgPDK1 would be an interesting topic to be investigated in the future.

In sum, we characterize a PDK1 from phytopathogen *F*. *graminearum*. FgPDK1 plays vital role in the regulation of the growth, toxin production, and pathogenicity of *F*. *graminearum*, which may be mediated by *Tri6*. Inhibition of PDK has been suggested as an attractive therapeutic approach for the development of anti-cancer drugs [53]. The PDK1 null mutant of yeast shows enhanced sensitivity to antibiotics (http://www.yeastgenome.org). Therefore, our current study not only shed new light on the physiological role of PDK1 in phytopathogen, but also provides a potential target for the control of FHB disease caused by *F*. *graminearum* infection.

## Supporting Information

S1 Table Oligonucleotide primers used in this study and their relevant characteristics. (DOCX)

S1 Fig The deletion of *FgPDK1* in *F*. *graminearum*. (A) *FgPDK1* fragments are denoted by large black and gray arrows, respectively. Annealing sites of PCR primers are indicated with arrows (see S1 Table for primer sequences). (B) PCR strategy to screen ΔFgPDK1 transformants. a: PCR performed with primer pair A7/R1; a 2.1-kb amplified fragment indicates ΔFgPDK1 integration at the left junction. b: PCR performed with primer pair F1/A8; 2.3-kb fragment amplification indicates ΔFgPDK1 integration at the right junction. c: PCR performed with primer pair A5/A6; a 0.5-kb amplification fragment indicates a wild type (PH-1) locus. Lane 1–6 indicate six replicates of transformants while lane 7 indicate wild type (PH-1). (C) A 485-bp *hph* fragment was used as a probe in Southern blot hybridization analyses. Genomic DNA preparations from the wild-type strain (PH-1), the *FgPDK1* deletion mutants (ΔFgPDK1-1 and ΔFgPDK1-2), and the complemented strain (ΔFgPDK1-C) were digested with Sac1.
(TIF)

S2 Fig Relative expression levels of four pigment-formation genes (*AurJ*, *AurF*, *AurO*, and *AurR2*) in wild-type strain (PH-1), the *FgPDK1* deletion mutants (ΔFgPDK1-1 and ΔFgPDK1-2), and the complemented strain (ΔFgPDK1-C). RNA was extracted from the mycelia of each strain after growth in potato dextrose broth for 2 days. Expression levels are relative to the amounts of cDNA in PH-1. Values are means±standard errors of three repeated experiments.
(TIF)

S3 Fig The micrograph of perithecium produced by *F*. *graminearum*. (A) *F*. *graminearum* produces black perithecium with ascospores inside. (B) Ascospores are released from broken perithecium.
(TIF)

## References

[1] Pronk JT, Yde Steensma H, Van Dijken JP. Pyruvate metabolism in *Saccharomyces cerevisiae*. Yeast 1996; 12: 1607–1633. 9123965 10.1002/(sici)1097-0061(199612)12:16<1607::aid-yea70>3.0.co;2-4

[2] Jeong JY, Jeoung NH, Park KG, Lee IK. Transcriptional regulation of pyruvate dehydrogenase kinase. Diabetes Metab J 2012; 36: 328–335. 10.4093/dmj.2012.36.5.328 23130316 PMC3486978

[3] Schulze A, Downward J. Flicking the Warburg switch-tyrosine phosphorylation of pyruvate dehydrogenase kinase regulates mitochondrial activity in cancer cells. Mol Cell 2011; 44: 846–848. 10.1016/j.molcel.2011.12.004 22195959

[4] Suthendra G, Michelakis ED. Pyruvate dehydrogenase kinase as a novel therapeutic target in oncology. Frontiers in Oncology 2013; 3: 38 10.3389/fonc.2013.00038 23471124 PMC3590642

[5] Marillia EF, Micallef BJ, Micallef M, Weninger A, Pedersen KK, Zou J, et al Biochemical and physiological studies of *Arabidopsis thaliana* transgenic lines with repressed expression of the mitochondrial pyruvate dehydrogenase kinase. J Exp Bot 2003; 54: 259–270. 12493853 10.1093/jxb/erg020

[6] Thelen JJ, Miernyk JA, Randall DD. Pyruvate dehydrogenase kinase from *Arabidopsis thaliana*: a protein histidine kinase that phosphorylates serine residues. Biochem J 2000; 349: 195–201. 10861228 10.1042/0264-6021:3490195 PMC1221137

[7] Li RJ, Hu ZY, Zhang HS, Zhan GM, Wang HZ, Hua W. Cloning and functions analysis of a pyruvate dehydrogenase kinase in *Brassica napus*. Plant Cell Rep 2011; 30: 1533–1540. 10.1007/s00299-011-1066-2 21461604

[8] Pereira RR, Castanheira D, Teixeira JA, Bouillet LE, Ribeiro EM, Tropia MM, et al Detailed search for protein kinase(s) involved in plasma membrane H+-ATPase activity regulation of yeast cells. FEMS Yeast Res 2015; 15. 10.1093/femsyr/fov003 25769530

[9] Gey U, Czupalla C, Hoflack B, Rodel G, Krause-Buchholz U. Yeast pyruvate dehydrogenase complex is regulated by a concerted activity of two kinases and two phosphatases. J Biol Chem 2008; 283: 9759–9767. 10.1074/jbc.M708779200 18180296

[10] Steensma HY, Tomaska L, Reuven P, Nosek J, Brandt R. Disruption of genes encoding pyruvate dehydrogenase kinases leads to retarded growth on acetate and ethanol in *Saccharomyces cerevisiae*. Yeast 2008; 25: 9–19. 17918780 10.1002/yea.1543

[11] Starkey DE, Ward TJ, Aoki T, Gale LR, Kistler HC, Geiser DM, et al Global molecular surveillance reveals novel Fusarium head blight species and trichothecene toxin diversity. Fungal Genet Biol 2007; 44: 1191–1204. 17451976 10.1016/j.fgb.2007.03.001

[12] Shen C-m, Hu Y-c, Sun H-y, Li W, Guo J-h, Chen H-g. Geographic distribution of trichothecene chemotypes of the *Fusarium graminearum* species complex in major winter wheat production areas of China. Plant Dis 2012; 96: 1172–1178. 10.1094/PDIS-11-11-0974-RE 30727056

[13] Sun HY, Zhu YF, Liu YY, Deng YY, Li W, Zhang AX, et al Evaluation of tebuconazole for the management of Fusarium head blight in China. Australas Plant Pathol 2014; 43: 631–638.

[14] Duan Y, Zhang X, Ge C, Wang Y, Cao J, Jia X, et al Development and application of loop-mediated isothermal amplification for detection of the F167Y mutation of carbendazim-resistant isolates in *Fusarium graminearum*. Sci Rep 2014; 4: 7094 10.1038/srep07094 25403277 PMC4235284

[15] Gu Q, Chen Y, Liu Y, Zhang C, Ma Z. The transmembrane protein FgSho1 regulates fungal development and pathogenicity via the MAPK module Ste50-Ste11-Ste7 in *Fusarium graminearum*. New Phytol 2015; 206: 315–328. 10.1111/nph.13158 25388878

[16] Correll JC, Klittich CJR, Leslie JF. Nitrate nonutilizing mutants of *Fusarium oxysporum* and their use in vegetative compatibility tests. Phytopathology 1987; 77: 1640–1646.

[17] Litsanov B, Kabus A, Brocker M, Bott M. Efficient aerobic succinate production from glucose in minimal medium with Corynebacterium glutamicum. Microb Biotechnol 2012; 5: 116–128. 10.1111/j.1751-7915.2011.00310.x 22018023 PMC3815278

[18] Chen Y, Li H, Chen C, Zhou M. Sensitivity of *Fusarium graminearum* to fungicide JS399-19:*In vitro* determination of baseline sensitivity and the risk of developing fungicide resistance. Phytoparasitica 36: 326–337.

[19] Zhang YJ, Fan PS, Zhang X, Chen CJ, Zhou MG. Quantification of *Fusarium graminearum* in harvested grain by real-time polymerase chain reaction to assess efficacies of fungicides on fusarium head blight, deoxynivalenol contamination, and yield of winter wheat. Phytopathology 2009; 99: 95–100. 10.1094/PHYTO-99-1-0095 19055440

[20] Marchler-Bauer A, Derbyshire MK, Gonzales NR, Lu S, Chitsaz F, Geer LY, et al CDD: NCBI's conserved domain database. Nucleic Acids Res 2015; 43: D222–226. 10.1093/nar/gku1221 25414356 PMC4383992

[21] Larkin MA, Blackshields G, Brown NP, Chenna R, McGettigan PA, McWilliam H, et al Clustal W and Clustal X version 2.0. Bioinformatics 2007; 23: 2947–2948. 17846036 10.1093/bioinformatics/btm404

[22] Kumar S, Dudley J, Nei M, Tamura K. MEGA: A biologist-centric software for evolutionary analysis of DNA and protein sequences. Brief Bioinform 2008; 9: 299–306. 10.1093/bib/bbn017 18417537 PMC2562624

[23] Zhang LG, Zhang Y, Li BC, Jia XJ, Chen CJ, Zhou MG. Involvement of *FgMad2* and *FgBub1* in regulating fungal development and carbendazim resistance in *Fusarium graminearum*. Plant Pathology 2015; 64: 1014–1028.

[24] Gao T, Zheng Z, Hou Y, Zhou M. Transcription factors spt3 and spt8 are associated with conidiation, mycelium growth, and pathogenicity in *Fusarium graminearum*. FEMS Microbiol Lett 2014; 351: 42–50. 10.1111/1574-6968.12350 24289742

[25] Liu SM, Chen Y, Yu JJ, Chen CJ, Wang JX, Zhou MG. Transfer of the *β-tubulin* gene of *Botrytis cinerea* with resistance to carbendazim into *Fusarium graminearum*. Pest Manag Sci 2010; 66: 482–489. 10.1002/ps.1897 20063268

[26] Maier FJ, Malz S, Losch AP, Lacour T, Schafer W. Development of a highly efficient gene targeting system for *Fusarium graminearum* using the disruption of a polyketide synthase gene as a visible marker. FEMS Yeast Res 2005; 5: 653–662. 15780665 10.1016/j.femsyr.2004.12.008

[27] Jiang J, Yun Y, Liu Y, Ma Z. *FgVELB* is associated with vegetative differentiation, secondary metabolism and virulence in *Fusarium graminearum*. Fungal Genet Biol 2012; 49: 653–662. 10.1016/j.fgb.2012.06.005 22713714

[28] Qiu J, Xu J, Yu J, Bi C, Chen C, Zhou M. Localisation of the benzimidazole fungicide binding site of *Gibberella zeae β2*-tubulin studied by site-directed mutagenesis. Pest Manag Sci 2011; 67: 191–198. 10.1002/ps.2050 21077124

[29] Duan Y, Ge C, Liu S, Chen C, Zhou M. Effect of phenylpyrrole fungicide fludioxonil on morphological and physiological characteristics of *Sclerotinia sclerotiorum*. Pestic Biochem Physiol 2013; 106: 61–67.

[30] Yang Y, Fan F, Zhuo R, Ma F, Gong Y, Wan X, et al Expression of the laccase gene from a white rot fungus in *Pichia pastoris* can enhance the resistance of this yeast to H_2_O_2_-mediated oxidative stress by stimulating the glutathione-based antioxidative system. Appl Environ Microbiol 2012; 78: 5845–5854. 10.1128/AEM.00218-12 22706050 PMC3406150

[31] Firstencel H, Butt TM, Carruthers RI. A fluorescence microscopy method for determining the viability of entomophthoralean fungal spores. J Invertebr Pathol 1990; 55: 258–264.

[32] Hu L, Zhou W, Yang J, Chen J, Yin Y, Shi Z. Cinnamaldehyde Induces PCD-Like death of *Microcystis aeruginosa* via reactive oxygen species. Water Air Soil Pollut 2011; 217: 105–113.

[33] Chen C, Wang J, Luo Q, Yuan S, Zhou M. Characterization and fitness of carbendazim-resistant strains of *Fusarium graminearum* (wheat scab). Pest Manag Sci 2007; 63: 1201–1207. 17955449 10.1002/ps.1449

[34] Di Pietro A, Garcia-MacEira FI, Meglecz E, Roncero MI. A MAP kinase of the vascular wilt fungus *Fusarium oxysporum* is essential for root penetration and pathogenesis. Mol Microbiol 2001; 39: 1140–1152. 11251832

[35] Kong W, Huang C, Chen Q, Zou Y, Zhang J. Nitric oxide alleviates heat stress-induced oxidative damage in *Pleurotus eryngii* var. *tuoliensis*. Fungal Genet Biol 2012; 49: 15–20. 10.1016/j.fgb.2011.12.003 22202810

[36] Proctor RH, Hohn TM, McCormick SP. Reduced virulence of *Gibberella zeae* caused by disruption of a trichothecene toxin biosynthetic gene. Mol Plant Microbe Interact 1995; 8: 593–601. 8589414 10.1094/mpmi-8-0593

[37] Jansen C, von Wettstein D, Schafer W, Kogel KH, Felk A, Maier FJ. Infection patterns in barley and wheat spikes inoculated with wild-type and trichodiene synthase gene disrupted *Fusarium graminearum*. Proc Natl Acad Sci U S A 2005; 102: 16892–16897. 16263921 10.1073/pnas.0508467102 PMC1283850

[38] Zhang S, Hulver MW, McMillan RP, Cline MA, Gilbert ER. The pivotal role of pyruvate dehydrogenase kinases in metabolic flexibility. Nutr Metab (Lond) 2014; 11: 10. 24520982 10.1186/1743-7075-11-10 PMC3925357

[39] Weraduwage SM, Micallef MC, Marillia EF, Taylor DC, Grodzinski B, Micallef BJ. Increased mtPDH activity through antisense inhibition of mitochondrial pyruvate dehydrogenase kinase enhances inflorescence initiation, and inflorescence growth and harvest index at elevated CO_2_ in *Arabidopsis thaliana*. Front Plant Sci 2016; 7: 95 10.3389/fpls.2016.00095 26904065 PMC4751281

[40] Randle PJ. Fuel selection in animals. Biochem Soc Trans 1986; 14: 799–806. 3536635 10.1042/bst0140799

[41] Hynes MJ, Szewczyk E, Murray SL, Suzuki Y, Davis MA, Sealy-Lewis HM. Transcriptional control of gluconeogenesis in *Aspergillus nidulans*. Genetics 2007; 176: 139–150. 17339216 10.1534/genetics.107.070904 PMC1893031

[42] Suzuki Y, Murray SL, Wong KH, Davis MA, Hynes MJ. Reprogramming of carbon metabolism by the transcriptional activators AcuK and AcuM in *Aspergillus nidulans*. Mol Microbiol 2012; 84: 942–964. 10.1111/j.1365-2958.2012.08067.x 22500966

[43] Belozerskaya TA, Gessler NN. Reactive oxygen species and the strategy of antioxidant defense in fungi: A review. Appl Biochem Microbiol 43: 506–515. 18038677

[44] Dickinson BC, Chang CJ. Chemistry and biology of reactive oxygen species in signaling or stress responses. Nat Chem Biol 2011; 7: 504–511. 10.1038/nchembio.607 21769097 PMC3390228

[45] Altintas A, Martini J, Mortensen UH, Workman CT. Quantification of oxidative stress phenotypes based on high-throughput growth profiling of protein kinase and phosphatase knockouts. FEMS Yeast Res 2016; 16: fov101. 26564984 10.1093/femsyr/fov101

[46] Newington JT, Rappon T, Albers S, Wong DY, Rylett RJ, Cumming RC. Overexpression of pyruvate dehydrogenase kinase 1 and lactate dehydrogenase A in nerve cells confers resistance to amyloid beta and other toxins by decreasing mitochondrial respiration and reactive oxygen species production. J Biol Chem 2012; 287: 37245–37258. 10.1074/jbc.M112.366195 22948140 PMC3481323

[47] Portillo F. Regulation of plasma membrane H(+)-ATPase in fungi and plants. Biochim Biophys Acta 2000; 1469: 31–42. 10692636 10.1016/s0304-4157(99)00011-8

[48] Merhej J, Richard-Forget F, Barreau C. Regulation of trichothecene biosynthesis in Fusarium: recent advances and new insights. Appl Microbiol Biotechnol 2011; 91: 519–528. 10.1007/s00253-011-3397-x 21691790

[49] Proctor RH, Hohn TM, McCormick SP, Desjardins AE. Tri6 encodes an unusual zinc finger protein involved in regulation of trichothecene biosynthesis in Fusarium sporotrichioides. Appl Environ Microbiol 1995; 61: 1923–1930. 7646028 10.1128/aem.61.5.1923-1930.1995 PMC167455

[50] Hohn TM, Beremand PD. Isolation and nucleotide sequence of a sesquiterpene cyclase gene from the trichothecene-producing fungus *Fusarium sporotrichioides*. Gene 1989; 79: 131–138. 2777086 10.1016/0378-1119(89)90098-x

[51] Seong K-Y, Pasquali M, Zhou X, Song J, Hilburn K, McCormick S, et al Global gene regulation by *Fusarium* transcription factors *Tri6* and *Tri10* reveals adaptations for toxin biosynthesis. Mol Microbiol 2009; 72: 354–367. 10.1111/j.1365-2958.2009.06649.x 19320833

[52] Nasmith CG, Walkowiak S, Wang L, Leung WWY, Gong Y, Johnston A, et al Tri6 is a global transcription regulator in the phytopathogen *Fusarium graminearum*. PLoS Pathog 2011; 7: e1002266 10.1371/journal.ppat.1002266 21980289 PMC3182926

[53] Zhang W, ZHang S-L, Hu X, Tam KY. Targeting tumor metabolism for cancer treatment: is pyruvate dehydrogenase kinases (PDKs) a viable anticancer target? Int J Biol Sci 2015; 11: 1390–1400. 10.7150/ijbs.13325 26681918 PMC4671996

