# Supplementary material for: Fusarium graminearum pyruvate dehydrogenase kinase 1 (FgPDK1) Is Critical for Conidiation, Mycelium Growth, and Pathogenicity
Source: PLoS One. 2016 Jun 24;11(6):e0158077. doi: 10.1371/journal.pone.0158077 (PMC4920349; doi:10.1371/journal.pone.0158077)
Supplement: S1 Table — (DOCX) [file pone.0158077.s001.docx]

**S1 Table. Oligonucleotide primers used in this study and their relevant characteristics**

| Primer | Sequence (5'-3') | Use |
| --- | --- | --- |
| A1 | GGACGTACACTGGACAGC | Amplify the left homologous arm of *FgPDK1* |
| A2 | CATTGATGGTTGACCTCCAGTAGCATACTGAGCCAGCA |  |
| A3 | CGAGGGCAAAGGAATAGAGTTACTGGCTTCATTTTTG | Amplify the right homologous arm of *FgPDK1* |
| A4 | GAGAAGGGTGACAAGCAG |  |
| A5 | CGGTATCAAGACGGAGTA | Amplify the partial *FgPDK1* and confirm whether *FgPDK1* replaced the  *hph* gene in *F. graminearum* |
| A6 | GAGATTCGGATGAAAGAG |  |
| A7 | GTCGGACATCGCATCTTA | Confirm whether the *hph* gene homologously replaced *FgPDK1* |
| A8 | AAGTCCCTTGACCCTAAC |  |
| A9 | ACTCACTATAGGGCGAATTGGGTACTCAAATTGGTTGGACGTACACTGGACAGC | Amplify the full-length *PDK1* gene |
| A10 | CACCACCCCGGTGAACAGCTCCTCGCCCTTGCTCACCTGCTGCTTATCCAAGTTTC |  |
| F1 | CCGCCTTGACCAGGGTGAGAT | Confirm whether the *hph* gene homologously replaced *FgPDK1* |
| R1 | GGTGTCGTCCATCACAGTTTGC |  |
| HTF | GGAGGTCAACACATCAATG | Amplify the *hph* gene |
| HTR | CTCTATTCCTTTGCCCTCG |  |
| P1 | GCAGACAGGAACGAGGAC | The 484-bp probe1 for the Southern blotting |
| P2 | CGGTTTCAGGCAGGTCTTG |  |
| P5 | AAAAAGCAGCCAAGGAGCAT | qRT-PCR primers for the *AurJ* gene |
| P6 | TTCTGATGACACGCTCCCGTA |  |
| P7 | ATCTTCAGTCTTGACCATCCC | qRT-PCR p imers for the *AurF* gene |
| P8 | TACCCAAGATGTTCTGGCAA |  |
| P9 | TCGGCACATCAGTATCTCCAA | qRT-PCR primers for the *AurO* gene |
| P10 | CAATACTATCGCCTGTCGCTT |  |
| P11 | AGGTCGTTGACACGGCAT | qRT-PCR primers for the *AurR_2_* gene |
| P12 | TGTGCCAGGAGTAAACTTTGA |  |
| P13 | TCTACCCACTCTACCAGTTCTT | qRT-PCR primers for the *FlbC* gene |
| P14 | CCTTGAAGAGACAGACTCAATGT |  |
| P15 | TGAGAAGGGCGAATGCTATG | qRT-PCR primers for the *Ren1* gene |
| P16 | GTTCTTCTCCTCGACACTGAAA |  |
| P17 | GAGTGTTTCATGCATGGCTACGTC | qRT-PCR primers for the *Tri5* gene |
| P18 | CTGAGCCTCCTTCACATCGTCC |  |
| P19 | TATCGAAAATTATATAACCACATC | qRT-PCR primers for the *Tri6* gene |
| P20 | CTGAGGGCATTCTGAGTAGCGACA |  |
