# Supplementary figures and images for: Fusarium graminearum pyruvate dehydrogenase kinase 1 (FgPDK1) Is Critical for Conidiation, Mycelium Growth, and Pathogenicity
Source: PLoS One. 2016 Jun 24;11(6):e0158077. doi: 10.1371/journal.pone.0158077 (PMC4920349; doi:10.1371/journal.pone.0158077)

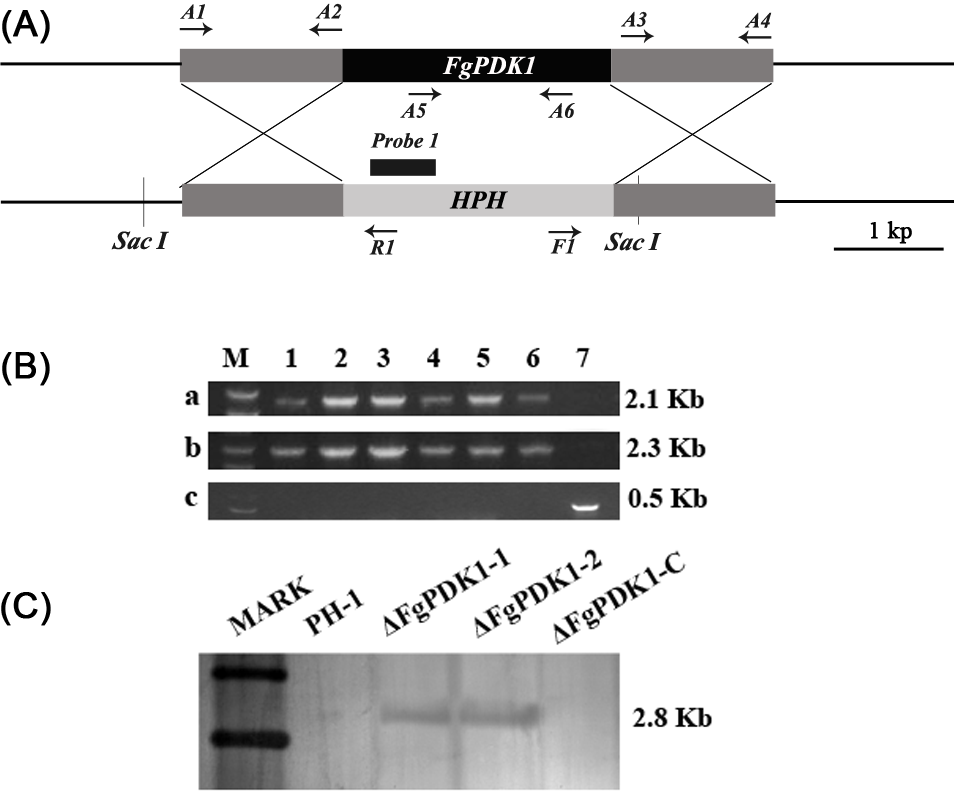

Supplement: S1 Fig — (A) FgPDK1 fragments are denoted by large black and gray arrows, respectively. Annealing sites of PCR primers are indicated with arrows (see S1 Table for primer sequences). (B) PCR strategy to screen ΔFgPDK1 transformants. a: PCR performed with primer pair A7/R1; a 2.1-kb amplified fragment indicates ΔFgPDK1 integration at the left junction. b: PCR performed with primer pair F1/A8; 2.3-kb fragment amplification indicates ΔFgPDK1 integration at the right junction. c: PCR performed with primer pair A5/A6; a 0.5-kb amplification fragment indicates a wild type (PH-1) locus. Lane 1–6 indicate six replicates of transformants while lane 7 indicate wild type (PH-1). (C) A 485-bp hph fragment was used as a probe in Southern blot hybridization analyses. Genomic DNA preparations from the wild-type strain (PH-1), the FgPDK1 deletion mutants (ΔFgPDK1-1 and ΔFgPDK1-2), and the complemented strain (ΔFgPDK1-C) were digested with Sac1. (TIF) [file pone.0158077.s002.tif]

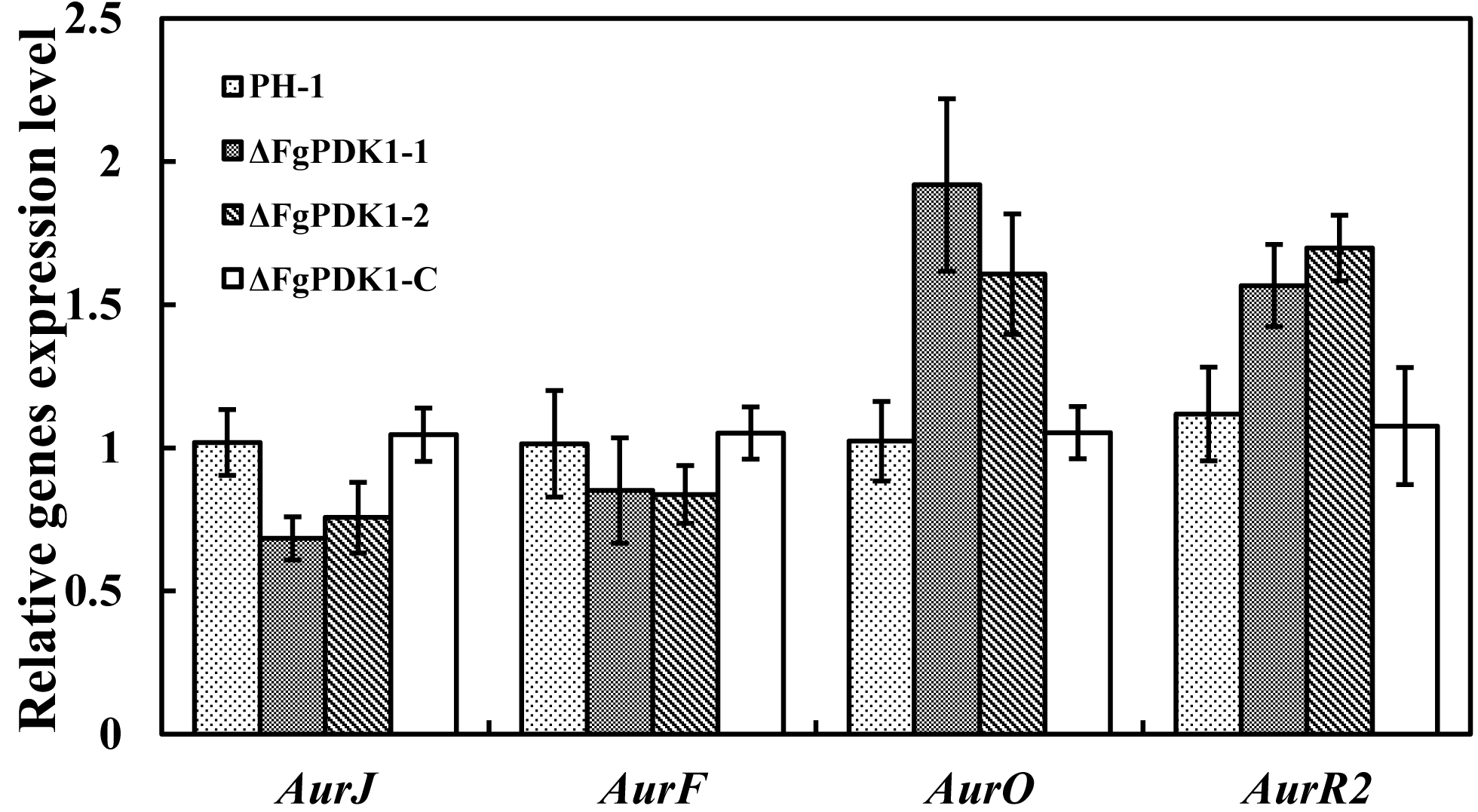

Supplement: S2 Fig — RNA was extracted from the mycelia of each strain after growth in potato dextrose broth for 2 days. Expression levels are relative to the amounts of cDNA in PH-1. Values are means±standard errors of three repeated experiments. (TIF) [file pone.0158077.s003.tif]

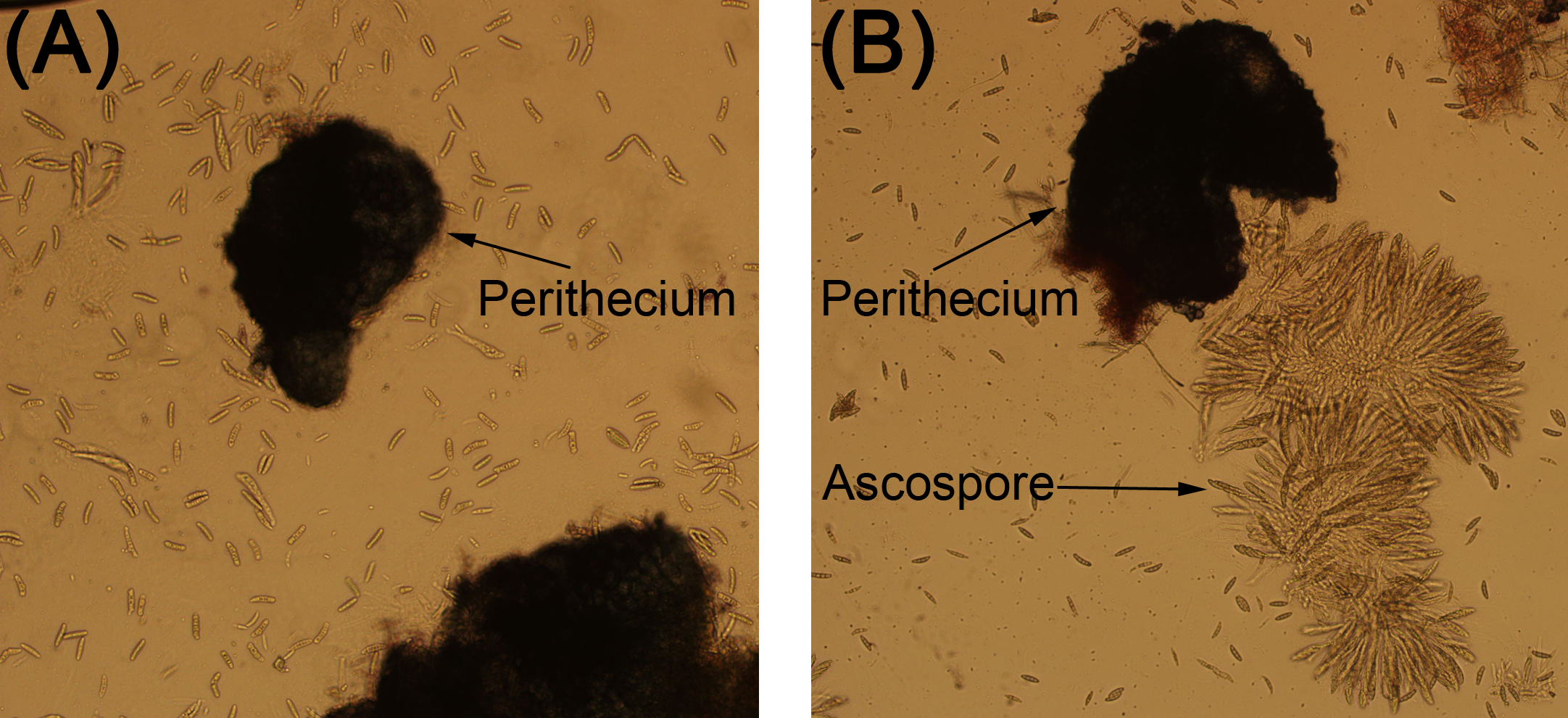

Supplement: S3 Fig — (A) F. graminearum produces black perithecium with ascospores inside. (B) Ascospores are released from broken perithecium. (TIF) [file pone.0158077.s004.tif]
